# Integrative Analyses of Circulating mRNA and lncRNA Expression Profile in Plasma of Lung Cancer Patients

**DOI:** 10.3389/fonc.2022.843054

**Published:** 2022-03-31

**Authors:** Haoran Li, Mingru Li, Haifa Guo, Guihu Lin, Qi Huang, Mantang Qiu

**Affiliations:** ^1^Department of Thoracic Surgery, Peking University People’s Hospital, Beijing, China; ^2^Department of Thoracic Surgery, Aerospace 731 Hospital, Beijing, China; ^3^The First Department of Thoracic Surgery, Beijing Chest Hospital, Capital Medical University, Beijing, China; ^4^Department of Thoracic Surgery, The First Affiliated Hospital of Zhengzhou University, Zhengzhou, China

**Keywords:** lung cancer, high-throughput sequencing, plasma RNA, cell-free RNAs, expression profiles

## Abstract

Circulating-free RNAs (cfRNAs) have been regarded as potential biomarkers for “liquid biopsy” in cancers. However, the circulating messenger RNA (mRNA) and long noncoding RNA (lncRNA) profiles of lung cancer have not been fully characterized. In this study, we profiled circulating mRNA and lncRNA profiles of 16 lung cancer patients and 4 patients with benign pulmonary nodules. Compared with benign pulmonary nodules, 806 mRNAs and 1,762 lncRNAs were differentially expressed in plasma of lung adenocarcinoma patients. For lung squamous cell carcinomas, 256 mRNAs and 946 lncRNAs were differentially expressed. A total of 231 mRNAs and 298 lncRNAs were differentially expressed in small cell lung cancer. Eleven mRNAs, 51 lncRNAs, and 207 canonical pathways were differentially expressed in lung cancer in total. Forty-five blood samples were collected to verify our findings *via* performing qPCR. There are plenty of meaningful mRNAs and lncRNAs that were found. MYC, a transcription regulator associated with the stemness of cancer cells, is overexpressed in lung adenocarcinoma. Transforming growth factor beta (TGFB1), which plays pleiotropic roles in cancer progression, was found to be upregulated in lung squamous carcinoma. MALAT1, a well-known oncogenic lncRNA, was also found to be upregulated in lung squamous carcinoma. Thus, this study provided a systematic resource of mRNA and lncRNA expression profiles in lung cancer plasma.

## Introduction

As the leading cause of cancer deaths, lung cancer is a serious threat to human health, accounting for over 1.8 million deaths in 2020 (1). Lung cancer consists of two histological subtypes: non-small cell carcinoma (NSCLC) and small cell lung carcinoma (SCLC) (2). As the mainly subtype, NSCLC, accounting for approximately 80% lung cancer, includes lung adenocarcinoma (LUAD), lung squamous cell carcinoma (LUSC), and large cell carcinoma. Although the relevant therapeutic strategies of surgery, chemotherapy, and target therapy for lung cancer have been improved over the past years, the prognosis is still unsatisfied (3). Due to the advanced stage of lung cancer at diagnosis, the early detection of the disease is most important for the promotion of patients’ prognosis.

The promising non-invasive method for early detection of lung cancer is the examination of specific biomarkers in blood (4). In 1948, cell-free nuclear acids [cfNA, such as DNA, messenger RNA (mRNA), and noncoding RNA] were described by Mandel and Metais for the first time (5). During the past decades, studies revealed that there are plenty of cfNAs in the blood of cancer patients (6). Interest is growing in the detection of cfRNAs in several cancers, such as hepatocellular carcinoma (7), breast cancer (8), and prostate cancer (9). During the progression of tumor, the cfRNA molecules in blood may be shed by the apoptosis and necrosis of cancer cells (6). In recent years, the cfRNA in plasma was thought to be a “liquid biopsy” for cancer diagnosis and prognosis (10). Although a series of studies have described the different expression panels of miRNAs (11, 12), few studies focused on the mRNA and lncRNA features in the plasma of lung cancers.

A further insight into the molecular characteristics can help us gain a better understanding of lung cancer and discovery of some novel biomarkers for early diagnosis and prognosis. High-throughput RNA sequencing provides a platform to analyze transcriptome comprehensively in various diseases, especially in cancers.

In our study, the mRNA and lncRNA expression profiles in the plasma of lung cancer patients were compared with those of lung hamartoma patients by whole transcriptome sequencing. Analysis of RNA-seq data revealed that a set of mRNAs and lncRNAs are differentially expressed in subtypes of lung cancer. Moreover, these results provide novel information on the comparation of lung cancer with benign lesion, which might be beneficial to search circulating candidates for early diagnosis of lung cancer.

## Materials and Methods

### Patients and Blood Samples

This study was approved by the Ethics Committee of the Peking University People’s Hospital, and informed consent was obtained from each participant. Blood samples were obtained from 20 patients that included patients with LUAD (lung adenocarcinoma, n=11), LUSC (lung squamous cell carcinoma, n=3), SCLC (small-cell lung cancer, n=2), and LUH (lung hamartoma, n=4) who underwent surgery in 2020 at the Department of Thoracic Surgery of Peking University People’s Hospital. Peripheral blood was collected before any treatment, and the 20 patients were confirmed by pathological diagnosis. Forty-five blood samples, including LUAD (15 samples), LUSC (15 samples), and healthy donor (15 samples), were collected to validate the results of differentially expressed mRNAs and lncRNAs.

### Sample Preparation, RNA Isolation, and Quantitative Real-Time PCR

Peripheral blood of each participants was collected before surgery using ethylenediaminetetraacetic acid (EDTA) tubes and immediately processed to isolate plasma. Blood samples were centrifuged at 1,600×*g* for 10 min, and then, supernatants were centrifuged at 16,000×*g* for 10 min. Plasma samples were stored at −80°C. Total RNA was isolated from the plasma using Trizol LS reagent (Invitrogen, CA, USA) separately. The RNA quality was checked for the RNA integrity number (RIN) by Bioanalyzer 2100 (Agilent, Santa Clara, CA, USA) and stored at −80°C. The procedure of reverse transcription followed the manufacturer’s protocol (TaKaRa, Shiga, Japan). Each quantitative polymerase chain reaction was performed in Applied Biosystem with a total reaction volume of 10 μl (Thermo Fisher, Waltham, MA, USA). Moreover, glyceraldehyde 3-phosphate dehydrogenase (GAPDH) was used as an internal control. The expression of mRNAs and lncRNAs were calculated using 2^−ΔΔCt^ methods. Relative fold change of each sample was calculated using the mean GAPDH expression of healthy donor group as reference. The primers used in this study are showed in **Supplementary Table S1**.

### Library Construction and RNA Sequencing

The libraries were constructed following the manufacture’s instruction of SMARTer Stranded Total RNA-Seq Kit v2(TaKaRa Bio USA, Mountain View, CA, USA) with 1–10 ng input RNA. In brief, (1) the purified RNA were fragmented at 94°C for 4 min in the first step of the cDNA synthesis. (2) The addition of Illumina adapters and indexes to single-stranded cDNA was finished by the first round PCR. (3) AMPure Beads was used to purify the amplified RNA-seq library. (4) Ribosomal cDNA was depleted with ZapR v2 and R-Probes v2. (5) The second round of PCR of 15 cycles was performed for the final RNA-seq library amplification. (6) The amplified RNA-seq library was purified again by immobilization onto AMPure beads. (7) Libraries were quantified with Qubit 3.0 (Thermo Fisher Scientific, Waltham, MA, USA). A yield >2 ng/μl was considered as sufficient material for further library validation and sequencing. Library size distribution was evaluated by running samples on the Agilent 2100 Bioanalyzer, with a local maximum at ~300–400 bp.

### RNA-Sequencing Data Mapping

The reads were first mapped to the UCSC Genome Browser database using Bowtie2 version 2.1.0 (13), and the gene expression level was further obtained by RSEM v1.2.15 (14). LNCipedia (http://www.lncipedia.org) was performed for lincRNA annotation, and Cufflinks was used to identify the different expression lncRNAs (15). Trimmed mean of M-values (TMM) was implemented to normalize the gene expression. Then, the edgeR program (16) was used for further differential expression analysis. Genes with *p* < 0.05 and more than 1.5-fold changes were considered to be differentially expressed.

### Functional Analysis of mRNAs and lncRNAs

The Gene Ontology (GO) category analyses (GO, http://www.geneontology.org/) (17) and the Kyoto Encyclopedia of Genes and Genomes (KEGG) molecular pathway analyses (KEGG; http://www.genome.ad.jp/kegg/) (18) were performed to understand the biological functions of differentially expressed mRNAs and lncRNAs. GO analysis for biological processes, cellular components, and molecular function were implemented using clusterProfile with *p*<0.05 as the cut-off value. Besides, a pathway enrichment analysis of the differentially expressed genes was performed using the pathways from Reactome database (19). To identify the functional roles of differentially expressed gene related to canonical pathways and upstream regulators, Ingenuity Pathway Analysis (IPA, www.ingenuity.com/) was performed, and Fisher’s exact test with false discovery rate (FDR) was used to identify the significance (*p*<0.05).

### Statistical Analysis

Comparisons between groups were analyzed with Student’s t-tests. The results were regarded as statistically significant at p < 0.05. The statistical analysis was performed using the SPSS 23.0 (IBM‐SPSS Inc., Chicago, IL, USA). All graphs were built using GraphPad Prism 8.0 software (GraphPad Software Inc., La Jolla, CA, USA).

## Results

### Baseline Clinical Characteristics

Based on the histopathological verification, a total of 20 plasma samples consisting of 16 lung cancers (including 11 adenocarcinoma, 3 squamous cell carcinoma, and 2 small cell lung cancer samples) and 4 matching negative control samples (lung hamartoma) were detected in this study. The detailed clinical characteristics of the four subgroups are summarized in **Supplementary Table S2**.

The overall data analysis flow of our study is shown in **Supplementary Figure S1**. Total RNA of four subgroups was extracted and subjected to library construction and RNA sequencing. After quality control, reads were mapped to genome to analyze the expression of mRNAs and lncRNAs using TopHat. Function enrichment analyses were used to predict potential biological functions of the differentially expressed genes.

### Expression Profiles of mRNAs and lncRNAs in Plasma of LUAD

**Figure 1:**
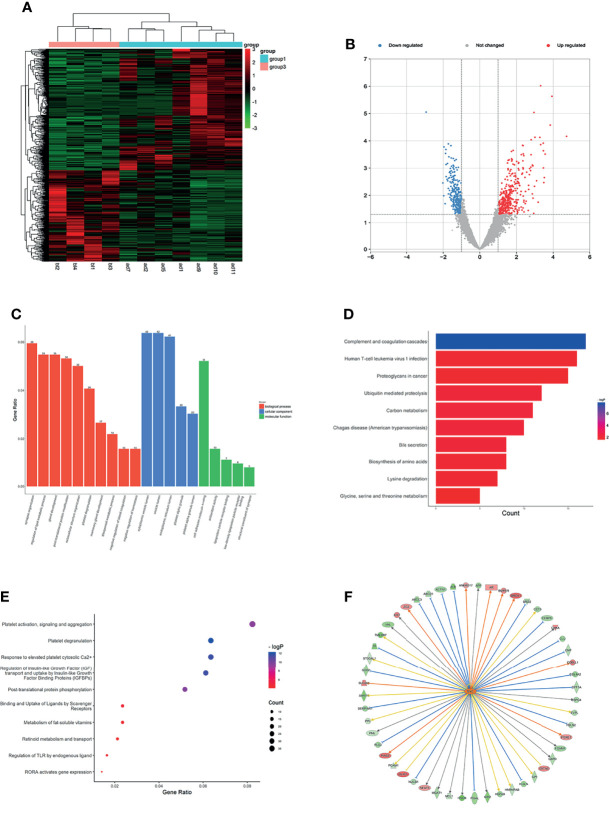
Expression profiles of mRNAs in plasma between LUAD patients and LUH negative controls. **(A)** Heatmap of differentially expressed mRNAs in LUAD patients and controls (group 1, LUAD plasma; group 3, LUH plasma). **(B)** Volcano plot of differentially expressed mRNAs (read, upregulated genes; green, downregulated genes). **(C)** Enrichment analysis of GO terms and pathways of the differentially expressed mRNAs, comprising biological process (BP), molecular function (MF), and cellular component (CC). **(D)** The top 10 KEGG terms for the differentially expressed mRNAs between two groups. **(E)** Reactome pathway analysis was performed to evaluate the underlying pathway. **(F)** Ingenuity pathway analysis predicted MYC as a major activation of transcription regulator in LUAD.

Using edgeR to identify the aberrantly expressed mRNAs based on the following criteria: ≥1.5−fold change (FC) upregulation or <1.5−fold change downregulation in expression plus p<0.05. A number of 5,685 mRNAs were detected, and a total of 806 differentially expressed mRNAs were identified in the peripheral blood (**Supplementary Table 3**), of which 459 mRNAs were upregulated and 347 mRNAs were downregulated (**Figure 1**). To visualize the differentially expressed mRNAs, the heatmap (**Figure 1A**) and volcano plot (**Figure 1B**) were analyzed.

Using Gene Oncology (GO) and Kyoto Encyclopedia of Genes and Genomes (KEGG) pathway analysis to investigate the gene functions. The different expressed mRNAs were significantly enriched in 637 biological processes (BP) terms, 92 molecular function (MF) terms, and 100 cellular component (CC) terms. The results showed that differentially expressed mRNAs were related to various biological processes, such as synapse organization, regulation of lipid metabolic process, post-translational protein modification, and extracellular structure organization signaling pathways (**Figure 1C**). GO analyses suggested that these genes were associated with molecular functions of cell adhesion molecule binding, antioxidant activity, lipoprotein particle receptor binding, and other important functions (**Figure 1C**). The top 3 CC terms were cytoplasmic vesicle lumen, endoplasmic reticulum lumen, and platelet alpha granule lumen, suggesting that these genes were mainly localized in the cytoplasm (**Figure 1C**). KEGG pathway analyses demonstrated that complement and coagulation cascades, human T-cell leukemia virus 1 infection, and proteoglycans in cancer were most enriched among the differentially expressed genes (**Figure 1D**). Reactome pathway analysis was performed to evaluate the underlying pathway, and platelet activation relevant pathways, regulation of insulin-like growth-factor binding proteins (IGFBPs), and post-translational protein phosphorylation were most enriched (**Figure 1E**).

To further understand the underlying molecular roles, IPA revealed the involvement of differentially expressed genes in several canonical pathways, including coagualtion system, epithelial adherens junction signaling, and phosphatase and tensin homolog (PTEN) signaling. IPA upstream regulator analysis revealed significant inhibition of several regulators involved in transcription regulator (NFE2L2 and STAT3), cytokine (OSM and IL6), growth factor (EGF and NRG1). The analysis also predicted a major activation of transcription regulator, MYC, which is a classical oncogene in LUAD, and MYC expression has been shown to be associated with the stemness of cancer cells (20). The network analysis showed that MYC could regulate 52 terms, including BRCA1, SLC1A1, and ITM2B (**Figure 1F**). The co-expression analysis revealed the relationship between these differentially expressed mRNAs and RN7SK and CPS1were the core genes (**Supplementary Figure S2A**).

**Figure 2:**
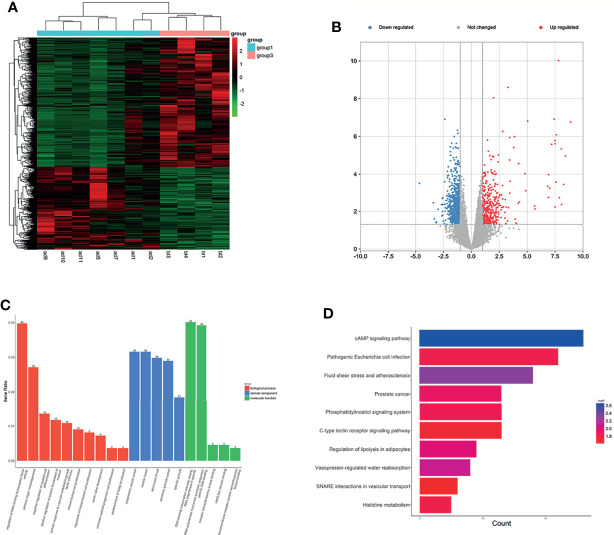
Expression profiles of lncRNAs in plasma between LUAD patients and LUH controls. **(A)** Heatmap of differentially expressed lncRNAs in LUAD patients and controls (group 1, LUAD plasma; group 3, LUH plasma). **(B)** Volcano plot of differentially expressed lncRNAs. **(C)** Enrichment analysis of GO terms and pathways of the differentially expressed lncRNAs. **(D)** KEGG pathway analysis of the differentially expressed mRNAs between two groups.

LncRNA expression profiles were normalized by TMM, and following criteria were employed for the differential expression analysis: p<0.05 and ≥1.5−fold upregulation or <1.5−fold downregulation in expression was performed. A total of 8,578 lncRNAs were characterized between LUAD plasma and LUH plasma and 1,762 lncRNAs (688 upregulation, 1,074 downregulation) were differentially expressed (**Supplementary Table S4**) and (**Figures 2A, B****)**. RAB23, UGDH, and LINC01322 were the top 3 downregulated lncRNA; IDH2-DT, CALML3, and LINC00982 were the top 3 upregulated lncRNA in LUAD plasma compared with LUH group. Previous studies have reported that the low expression of LINC00982 was associated with pathway alteration and poor patient survival of LUAD (21). The roles of LINC01322 and CALML3 in cancers were also revealed by previous studies (22, 23).

The functions of differentially expressed lncRNAs were predicted by GO and KEGG pathway annotations of their cis-regulated genes. GO analysis indicated that targets were enriched in 10 biological process (BP) terms and five molecular function (MF) terms. The top 3 BP terms were regulation of DNA-binding transcription factor activity, sensory organ morphogenesis, and negative regulation of myeloid cell differentiation. The CC terms reminded that these targets were localized to the cytoplasm. Besides, the enrichment of MF terms mainly associated with DNA-binding transcription activator activity (**Figure 2C**). According to KEGG pathway analyses, the differentially expressed lncRNAs are involved in some cancer-related pathways, and cAMP signaling pathway, pathogenic *Escherichia coli* infection, and fluid shear stress and atherosclerosis were most enriched (**Figure 2D**). The co-expression analysis revealed the relationship between these differentiallyexpressed lncRNAs, and lnc-IDS-8:1 and LINC00887:4 were the core genes (**Supplementary Figure S2B**).

### Expression Profiles of mRNAs and lncRNAs in Plasma of LUSC

**Figure 3:**
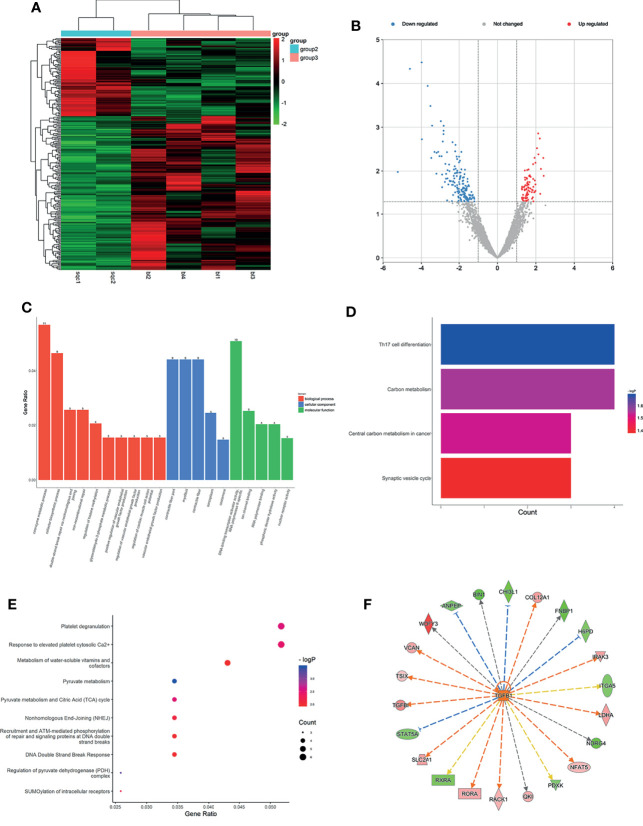
Expression profiles of mRNAs in plasma between LUSC patients and LUH negative controls. **(A)** Heatmap of differentially expressed mRNAs in LUSC patients and controls (group 2, LUSC plasma; group 3, LUH plasma). **(B)** Volcano plot of differentially expressed mRNAs (read, upregulated genes; green, downregulated genes). **(C)** Enrichment analysis of GO terms and pathways of the differentially expressed mRNAs. **(D)** The top 10 KEGG terms for the differentially expressed mRNAs between two groups. **(E)** Reactome pathway analysis was performed to evaluate the underlying pathway. **(F)** Ingenuity pathway analysis predicted TGFB1 as a major activation of transcription regulator in LUSC.

With thresholds of log2 FC>1.5 and p<0.05, a number of 3,907 mRNAs were detected, and a total of 170 downregulated (such as CTAGE8, WDFY3, and LSM8) and 86 upregulated mRNAs (such as SLC38A10, FAM120A, and PRR12) between LUSC and LUH were identified in the peripheral blood (**Supplementary Table S5**). The different mRNAs are displayed in a heatmap (**Figure 3A**) and volcano plot (**Figure 3B**), and the results indicated that mRNAs were obviously distinguishable between the two groups.

With the cutoff as p<0.05, the differentially expressed mRNAs were significantly enriched in 125 GO terms (78 in BP terms, 36 in MF terms, and 11 in CC terms). GO BP terms showed coenzyme metabolic process, regulation of histone methylation, and regulation of vascular endothelial growth factor production were most related (**Figure 3C**). CC terms indicated that products of these genes were mostly located at contractile fiber part and myofibril (**Figure 3C**). As for MF terms, the cellular activities were related to DNA-binding transcription activator activity, ion channel binding, and nuclear receptor activity (**Figure 3C**). KEGG pathway analyses showed that differentially expressed mRNAs were significantly involved in 23 pathways, and Th17 cell differentiation, central carbon metabolism in cancer, and synaptic vesicle cycle were most enriched among them (**Figure 3D**). Reactome pathway analyses indicated that these targets were significantly enriched in platelet degranulation relevant pathways, regulation of pyruvate metabolism, and recruitment, and ATM-mediated phosphorylation of repair and signaling proteins at DNA double-strand breaks pathways were most enriched (**Figure 3E**). Using IPA, the most significant canonical pathways on the differentially expressed genes were revealed, such as DNA double-strand break repair, oncostatin M signaling, and Wnt pathway. Besides, the analysis of the upstream regulators indicated that several significant inhibition regulators involved in transcription regulator (TP53 and EOMES), miRNA (mir-155, mir-25), and ligand-dependent nuclear receptor (NR3C2). A biological network predicted a major activation of growth factor, Transforming growth factor beta (TGFB1) (**Figure 3F**) is an important member of the transforming growth factor beta (TGF-β) family and plays pleiotropic roles in cancer progression (24). The co-expression analysis revealed the relationship between these differentially expressed mRNAs, and ZNF629 was the core gene (**Supplementary Figure S2C**).

**Figure 4:**
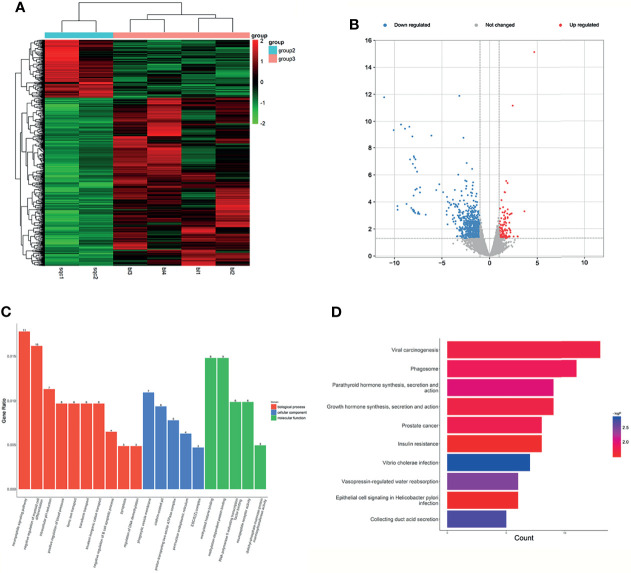
Expression profiles of lncRNAs in plasma between LUSC patients and negative controls. **(A)** Heatmap of differentially expressed lncRNAs in LUSC patients and LUH patients (group 2, LUSC plasma; group 3, LUH plasma). **(B)** Scatter plot of differentially expressed lncRNAs. **(C)** Enrichment analysis of GO terms and pathways of the differentially expressed lncRNAs. **(D)** KEGG pathway analysis of the differentially expressed mRNAs between two groups.

Based on the above screening criteria in *Section 2.1*, the expressed lncRNAs detected by edgeR in matched LUSC and LUH plasmas showed distinct expression patterns. A total of 7,462 lncRNAs were characterized, and 946 were differentially expressed (242 upregulation and 704 downregulation) in plasmas with LUSC compared with negative controls (p<0.05, fold change≥1.5 or <1.5) (**Supplementary Table S6**). Heatmap and volcano plots for the expression of these lncRNAs are shown in **Figures 4A, B**. The top 3 downregulated lncRNAs in LUSC were H2BFWT, MBTD1, and MALAT1, and the top 3 upregulated lncRNAs were Linc01663, TFF3, and SNRNP35. MALAT1, as a highly conserved lncRNA in mammals (25), is related to cancer development and progression (26). In addition, Weber et al. have reported MALAT1 could be detected in peripheral blood and serve as a promising biomarker for early diagnosis of NSCLC (27).

In the GO and KEGG processes, the enrichment analysis was performed on the function of lncRNA aided by its regulated gene. As shown in **Figure 4C**, the top rank of differentially expressed lncRNAs functions is listed. The impact on BP, such as regulation of DNA demethylation, transferrin transport, and pyroptosis process involved in development, indicated that these lncRNAs have potential biological functions, especially in tumor progression (28, 29). The top 3 MF terms were methylation-dependent protein binding, RNA polymerase II activating transcription factor binding, and protein tyrosine kinase binding, and the CC terms reminded these targets were localized to the cytomembrane and endoplasmic reticulum. In terms of KEGG pathway analyses, the results suggested that viral carcinogenesis signaling pathway, phagosome and parathyroid hormone synthesis, section, and action were most enriched among the differentially expressed genes (**Figure 4D**). The co-expression analysis revealed the relationship between these differentially expressed lncRNAs, and lnc-SRGAP2C-16:1 and lnc-FAM86B2-58:12 were the core genes (**Supplementary Figure S2D**).

### Expression Profiles of mRNAs and lncRNAs in Plasma of SCLC

**Figure 5:**
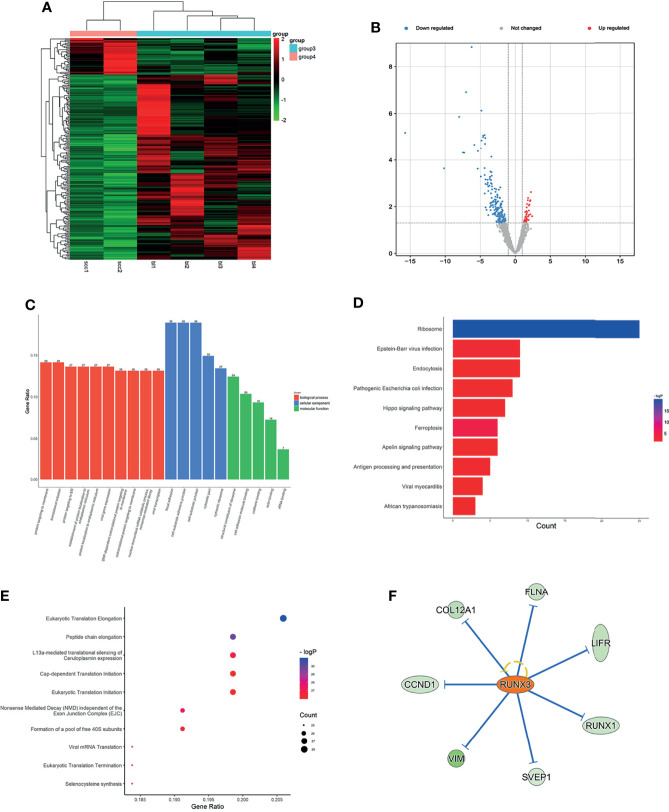
Expression profiles of mRNAs in plasma between SCLC patients and LUH negative controls. **(A)** Heatmap of differentially expressed mRNAs in SCLC patients and controls (group 4, SCLC plasma; group 3, LUH plasma). **(B)** Volcano plot of differentially expressed mRNAs (read, upregulated genes; green, downregulated genes). **(C)** Enrichment analysis of GO terms and pathways of the differentially expressed mRNAs. **(D)** The top 10 KEGG terms for the differentially expressed mRNAs between two groups. **(E)** Reactome pathway analysis was performed to evaluate the underlying pathway. **(F)** Ingenuity pathway analysis predicted RUNX3 as a major activation of transcription regulator in SCLC.

The RNA-seq results showed that a number of 3,284 mRNAs were detected, and 231 mRNAs in the SCLC group were significantly different from those in the LUH group (thresholds of log2 FC>1.5 and p<0.05) (**Supplementary Table S7**), of which 38 mRNAs were upregulated and 193 mRNAs were downregulated (**Figures 5A, B****)**.

Gene Ontology (GO) analyses revealed that the top 3 associated pathways of molecular functions were structural constituent of ribosome, cell adhesion molecule binding, and cadherin binding (**Figure 5C**). GO cellular component analyses indicated that products of these genes were mainly associated with cell adhesion. In terms of GO biological processes, differentially expressed mRNAs functions affected the protein targeting to membrane, protein localization to endoplasmic reticulum, and nuclear-transcribed mRNA catabolic process. The KEGG pathway analysis results are shown in **Figure 5C**. For the target genes of differentially expressed mRNAs, Hippo signaling pathway, ferroptosis, and Apelin signaling pathway were the most significant pathways for enrichment (**Figure 5D**). Reactome analyses showed that eukaryotic translation elongation pathways, peptide chain elongation and L13a-mediated translational silencing of Ceruloplasmin expression were most enriched (**Figure 5E**).

We further explored the canonical pathway analysis by IPA. The result revealed the involvement of several canonical pathways, including EIF2 signaling, mTOR signaling, and iron homeostasis signaling pathway. IPA upstream regulator analysis predicted a series of regulators involved in transcription regulator (MYCN and NFE2L2), growth factor (TGFB1 and NRG1), and transporter (SYVN1). In the network analysis, it can be observed that a major activation of transcription regulator, RUNX3, has a close interaction with a series of factors, such as CCND1, LIFR, COL12A1 (**Figure 5F**). The co-expression analysis revealed the relationship between these differentially expressed mRNAs, and RNA28S5 was the core gene (**Supplementary Figure S2E**).

**Figure 6:**
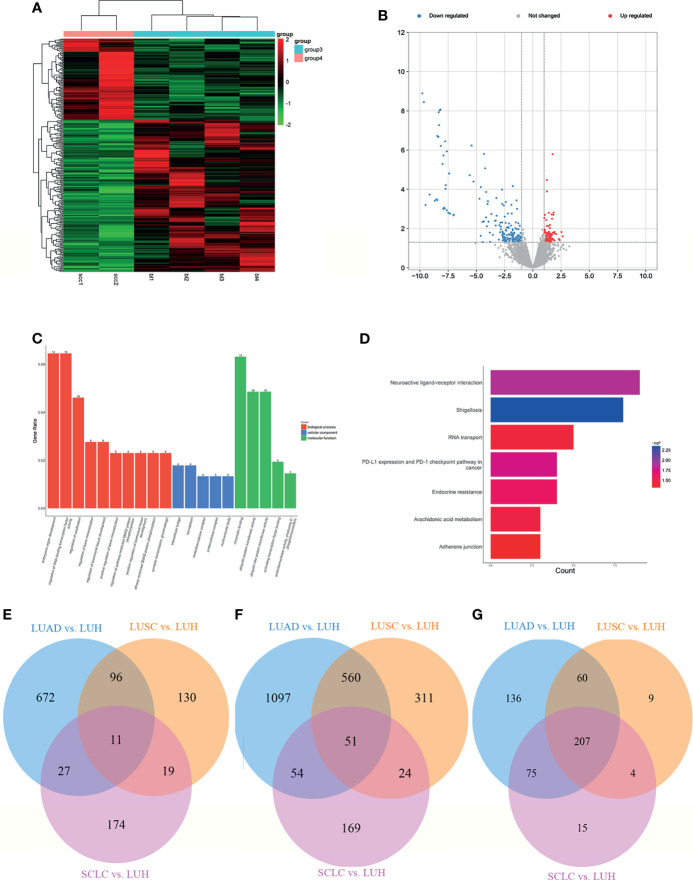
Expression profiles of lncRNAs in plasma between SCLC patients and negative controls. **(A)** Heatmap of differentially expressed lncRNAs in SCLC patients and LUH patients (group 4, SCLC plasma; group 3, LUH plasma). **(B)** Volcano plot of differentially expressed lncRNAs. **(C)** Enrichment analysis of GO terms and pathways of the differentially expressed lncRNAs. **(D)** KEGG pathway analysis of the differentially expressed mRNAs between two groups. Venn diagram showed the common significant mRNAs **(E)**, lncRNAs **(F)**, and canonical pathways **(G)** in three groups (LUAD, lung adenocarcinoma; LUSC, lung squamous cell carcinoma; SCLC, lung small cell cancer; LUH, lung hamartoma).

As for lncRNA expression profiles, the screening criteria are listed in *Section 2.1*.* A* total of 298 differentially expressed lncRNAs were finally characterized in the plasma of patients with SCLC, of which 104 lncRNAs were upregulated and the remaining lncRNAs were downregulated (**Supplementary Table S8**). Heatmap (**Figure 6A**) and volcano plot (**Figure 6B**) were used to analyze the statistical significance of differently expressed lncRNAs between the two groups. Several significant differently expressed lncRNAs have been reported in previous studies. The downregulation of LINC01537 and TMEM106A was observed in lung cancer development, which was involved in tumor metabolic reprogramming or EMT (30, 31).

The functional enrichment of GO and KEGG analysis is shown in **Figures 6C, D**. In BP terms, regulation of embryonic organ development, regulation of DNA-binding transcription factor activity, and regulation of bone mineralization involved in development were most enriched. The cis-regulated genes of differently expressed lncRNAs were mainly associated with intercellular bridge and sarcoplasm, according to the cellular component analysis. In addition, the most enriched MF terms, such as chromatin binding, ubiquitin−protein transferase activity, and activating transcription factor binding indicated that these lncRNAs may participate in ubiquitination or protein binding in post-transcription control. In terms of KEGG pathway analyses, the results suggested that RNA transport, PD-L1 expression, and PD-1 checkpoint pathway in cancer were most enriched among the differentially expressed genes. The co-expression analysis revealed the relationship between these differentially expressed lncRNAs, and lnc-CCNB1IP1-1:4 and lnc-WDR38-1:1 were the core genes (**Supplementary Figure S2F**).

### Common Dysregulated mRNAs and lncRNAs in Lung Cancer

We further investigated the common mRNAs of differential genes among three groups above. There are 11 common mRNAs in total (**Figure 6E**), including CEP250 (centrosomal protein 250 kDa), ZNF891 (zinc finger protein 891), NFAT5 (nuclear factor of activated T cells 5, tonicity-responsive), SLC2A1 (solute carrier family 2 member 1), TRIM38 (tripartite motif containing 38), PDE4A (phosphodiesterase 4A, cAMP-specific), GLYCTK (glycerate kinase), AFF2 (AF4/FMR2 family, member 2), WNK3 (WNK lysine-deficient protein kinase 3), BRWD3 (bromodomain and WD repeat domain containing 3), and ZCCHC2 (zinc finger, CCHC domain containing 2).

Then, we found the common lncRNAs among these three groups, and there are 51 lncRNAs in total (**Figure 6F**). Intriguingly, some lncRNAs were generated from common mRNAs above, such as lnc-TRIM38-2:2. Finally, we investigated the common canonical pathways, and 207 pathways were found (**Figure 6G**). The result of several canonical pathways includes molecular mechanisms of cancer, role of tissue factor in cancer, STAT3 pathway, PI3K/AKT signaling, and so on, which are vital for the progression of cancers.

### Validation of mRNAs and lncRNAs in LUAD and LUSC

**Figure 7:**
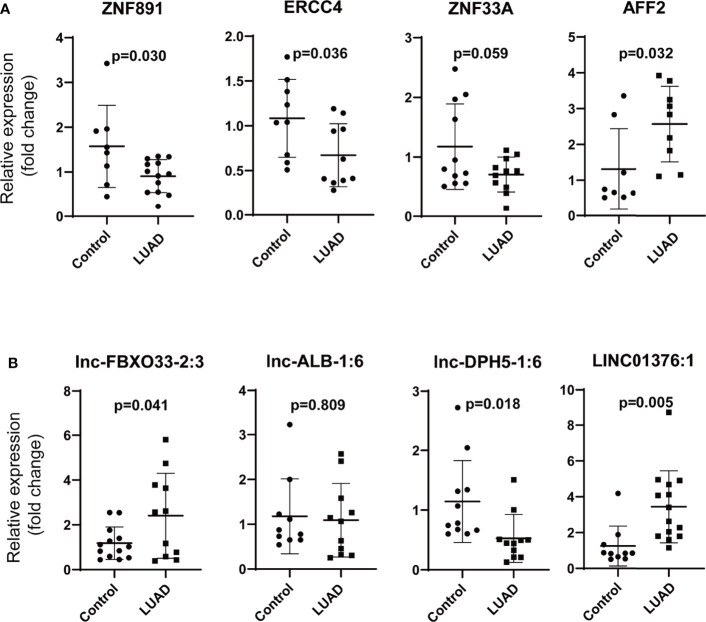
Validation of the differentially expressed mRNAs and lncRNAs in LUAD. **(A)** Validation of the differentially mRNAs (ZNF891, ERCC4, ZNF33A, and AFF2), **(B)** validation of the differentially lncRNAs (lnc-FBXO33-2:3, lnc-ALB-1:6, lnc-DPH5-1:6, and LINC01376:1) (LUAD, lung adenocarcinoma; ZNF891, zinc finger protein 891; ERCC4, excision repair cross-complementation group 4; ZNF33A, zinc finger protein 33A; AFF2, AF4/FMR2 family, member 2; relative fold change of each sample was calculated using the mean GAPDH expression of control group as reference).

We chose ZNF891, ERCC4 (excision repair cross-complementation group 4), ZNF33A (zinc finger protein 33A), AFF2 (AF4/FMR2 family, member 2), and lnc-ALB-1:6, lnc-DPH5-1:6, and LINC01376:1 to verify our findings in LUAD. The results of ZNF891, ZNF33A, ERCC4, and lnc-DPH5-1:6 were in accordance with the findings (**Figure 7**). However, the expression of AFF2, lnc-FBXO33-2:3, and LINC01376:1 was opposite to the previous findings, which may be due to the relatively small number of validation samples. Furthermore, no significant difference was observed for lnc-ALB-1:6.

**Figure 8:**
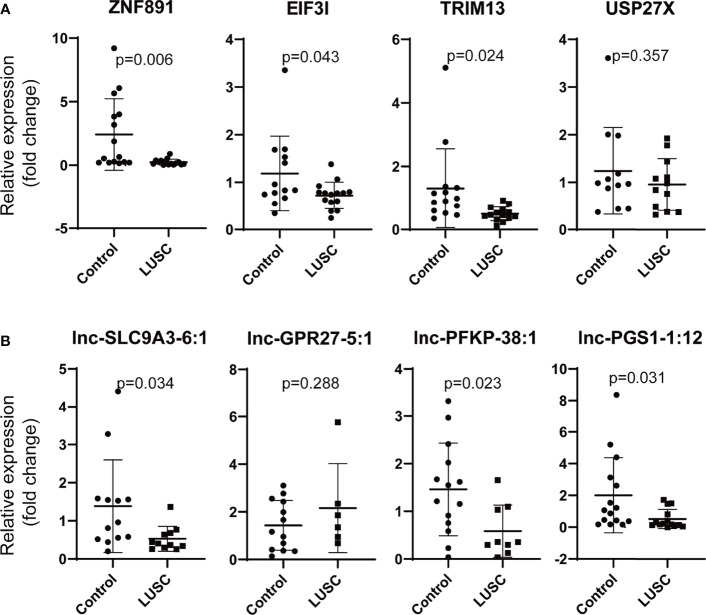
Validation of the differentially mRNAs and lncRNAs in LUSC. **(A)** Validation of the differentially expressed mRNAs (ZNF891, ERCC4, ZNF33A and AFF2), **(B)** validation of the differentially expressed lncRNAs (lnc-FBXO33-2:3, lnc-ALB-1:6, lnc-DPH5-1:6, and LINC01376:1) (LUSC, lung squamous cell carcinoma; ZNF891, zinc finger protein 891; EIF3I, eukaryotic translation initiation factor 3, subunit I; TRIM13, tripartite motif containing 13, USP27X, ubiquitin specific peptidase 27, X-linked; relative fold change of each sample was calculated using the mean GAPDH expression of control group as reference).

Moreover, ZNF891, EIF3I (eukaryotic translation initiation factor 3, subunit I), TRIM13 (tripartite motif containing 13), USP27X (ubiquitin specific peptidase 27, X-linked), lnc-SLC9A3-6:1, lnc-GPR27-5:1, lnc-PFKP-38:1, and lnc-PGS1-1:12 were selected to verify our findings in LUSC. ZNF891, EIF3I, TRIM13, lnc-SLC9A3-6:1, lnc-PFKP-38:1, and lnc-PGS1-1:12 were downregulated as we supposed (**Figure 8**). However, no significant difference was observed for USP27X or lnc-GPR27-5:1.

## Discussion

In the present study, the expression profiles of mRNAs and lncRNAs in plasma samples from lung cancer patients and benign pulmonary disease patients were compared based on RNA sequencing. The results indicated the different types of lung cancer presented distinguishing features of mRNAs and lncRNAs in the plasma of patients. Using a series gene function analysis, these different expression RNAs may be involved into various physiological processes, especially in cancer progression, which suggested the potential value of “liquid biopsy” in cancer diagnosis.

Tumor-derived mRNAs exist abundantly in blood and other biological fluids, which are related to tumorigenesis and progression (32). March-Villalba et al. found the that hTERT mRNA levels in plasma were associated with clinicopathological parameters of prostate cancer, which performed a better diagnostic and prognostic accuracy than the PSA assay (33). MiRNAs are a class of single-stranded non-coding RNAs, which are 18–23 nt in size and widely expressed in eukaryotes. The biological function of miRNAs is involved in the regulation of downstream target genes at the post-transcriptional level. Previous studies have proved the close relationship between miRNA abnormal expression patterns and tumorigenesis (34). Moreover, different panels of miRNAs in blood and other biological fluids presented specific expression in some cancers (35). Ying et al. described a five-miRNA panel for early detection of lung cancer with a 90.7% specificity (36). Compared with miRNAs, lncRNA is a novel type of non-protein coding transcripts, which is longer than 200 nucleotides (37). Emerging evidence indicated that lncRNAs are not “transcriptional noise” and play a crucial role in regulating chromatin dynamics, gene expression, growth, differentiation, and development (38). A large number of lncRNAs are involved in oncogenic process and tumor metastasis, which implied the potential promise for lncRNAs as novel biomarkers in cancers. Studies found that miRNAs and lncRNAs were stably presented in blood, and they showed a closed expression pattern relationship between primary tumors and plasmas of patients (32).

In our study, compared with lung hamartoma, 806 mRNAs and 1762 lncRNAs were significantly differentially expressed in patients with LUAD; 256 mRNAs and 946 lncRNAs were significantly differentially expressed in patients with LUSC. SCLC, accounting for 15% of lung cancers, is aggressive at early stage with a tendency of widespread metastases (39). SCLC is rarely resected by surgery, and the tissue is insufficient for further molecular investigation. Thus, exploring SCLC serological characteristics is needed. Our analysis indicated the expression features of mRNAs and lncRNAs in the plasma of SCLC patients.

eIF3i, also called p36 and eIF3β, is located at chromosome 1p35.2 as a putative subunit of eIF3 (eukaryotic initiation factor 3) (40). It has been reported that eIF3i plays an important role in pre-initiation complex formation and mRNA translation initiation (41). Ahlemann et al. demonstrated that *in vitro* eIF3i overexpression activated the mTOR signals and promoted the mRNA translation process and protein synthesis (42). Importantly, studies indicated that eIF3i is a proto-oncogene, and its expression has been proven to be upregulated in a series of cancers (43–45). The ectopic eIF3i overexpression is associated with the transformation of intestinal epithelial cells, and its level contributes to colon oncogenesis by upregulating the synthesis of cyclooxygenase-2 and activating the Wnt/β-catenin signaling pathway (45). In this study, eIF3i was significantly upregulated in the plasma of LUSC patients. Previous studies have not reported the role of eIF3i in lung cancer; the results of this study may remind that eIF3i serves as a potential biomarker for LUSC.

PDE4 enzymes are members of cyclic nucleotide phosphodiesterases (PDEs) family, and PDE4A is a subtype of PDF4 that regulates the cAMP level by promoting the degradation of cAMP to AMP (46). PDE4A has been reported to be involved in the regulation of tumor suppressor genes in tumors and hematological malignancies (47, 48). In line with previous observation, our finding revealed that PDE4A was overexpressed in SCLC plasma. This may be caused by the regulation of PDE4A in VEGF-mediated epithelial-to-mesenchymal transition (EMT) during tumor progression (49).

MALAT1 (metastasis associated lung adenocarcinoma transcript 1) is more than 8,500 nt and located at chromosome 11q13 (50). It was first characterized in NSCLC and played an important role in the prediction of metastasis (51). The highly conserved MALAT1 attracted the attention of researchers, and a series of studies have shown that MALAT1 was associated closely with tumor proliferation and invasion by interacting with several famous cancer-related signaling pathways (26, 52). Considering our results, we provide further evidence that overexpression of MALAT1 in plasma of LUSC patients may serve as a potential biomarker for diagnosis or treatment evaluation of LUSC.

In conclusion, our study performed a systematic description of mRNA and lncRNA profiles from the plasma of lung cancer patients and elucidated their functional modes based on a series of analysis. The current results suggest the potential value of cell-free RNAs for non-invasive “liquid biopsy.” These significant plasma biomarkers could be further explored for the diagnosis and prognosis of lung cancers.

## Data Availability Statement

The raw data supporting the conclusions of this article will be made available by the authors, without undue reservation.

## Author Contributions

MQ and QH: study concept and design. HL, ML, and HG: acquisition and analysis of the data. HL, GL, MQ, and QH: drafting and revising of the manuscript. All authors contributed to the article and approved the submitted version.

## Conflict of Interest

Author ML and GL are employed by Medical and Health of China Aerospace and Industry Co, Ltd. The remaining authors declare that the research was conducted in the absence of any commercial or financial relationships that could be construed as a potential conflict of interest.

## Publisher’s Note

All claims expressed in this article are solely those of the authors and do not necessarily represent those of their affiliated organizations, or those of the publisher, the editors and the reviewers. Any product that may be evaluated in this article, or claim that may be made by its manufacturer, is not guaranteed or endorsed by the publisher.

## Supplementary Material

Supplementary Figure 1 The overall data analysis flow. LUAD, lung adenocarcinoma; LUSC, lung squamous cell carcinoma; SCLC, small cell lung cancer; LUH, lung hamartoma.

Supplementary Figure 2 The co-expression of differentially mRNAs and lncRNAs in the study. The co-expression of mRNAs **(A)** and lncRNAs **(B)** in LUAD and LUH. The co-expression of mRNAs **(C)** and lncRNAs **(D)** in LUSC and LUH; The co-expression of mRNAs **(E)** and lncRNAs **(F)** in SCLC and LUH. (LUAD, lung adenocarcinoma; LUSC, lung squamous cell carcinoma; SCLC, small cell lung cancer; LUH, lung hamartoma.)
