# Supplementary figures and images for: Integrative Analyses of Circulating mRNA and lncRNA Expression Profile in Plasma of Lung Cancer Patients
Source: Front Oncol. 2022 Mar 31;12:843054. doi: 10.3389/fonc.2022.843054 (PMC9008738; doi:10.3389/fonc.2022.843054)

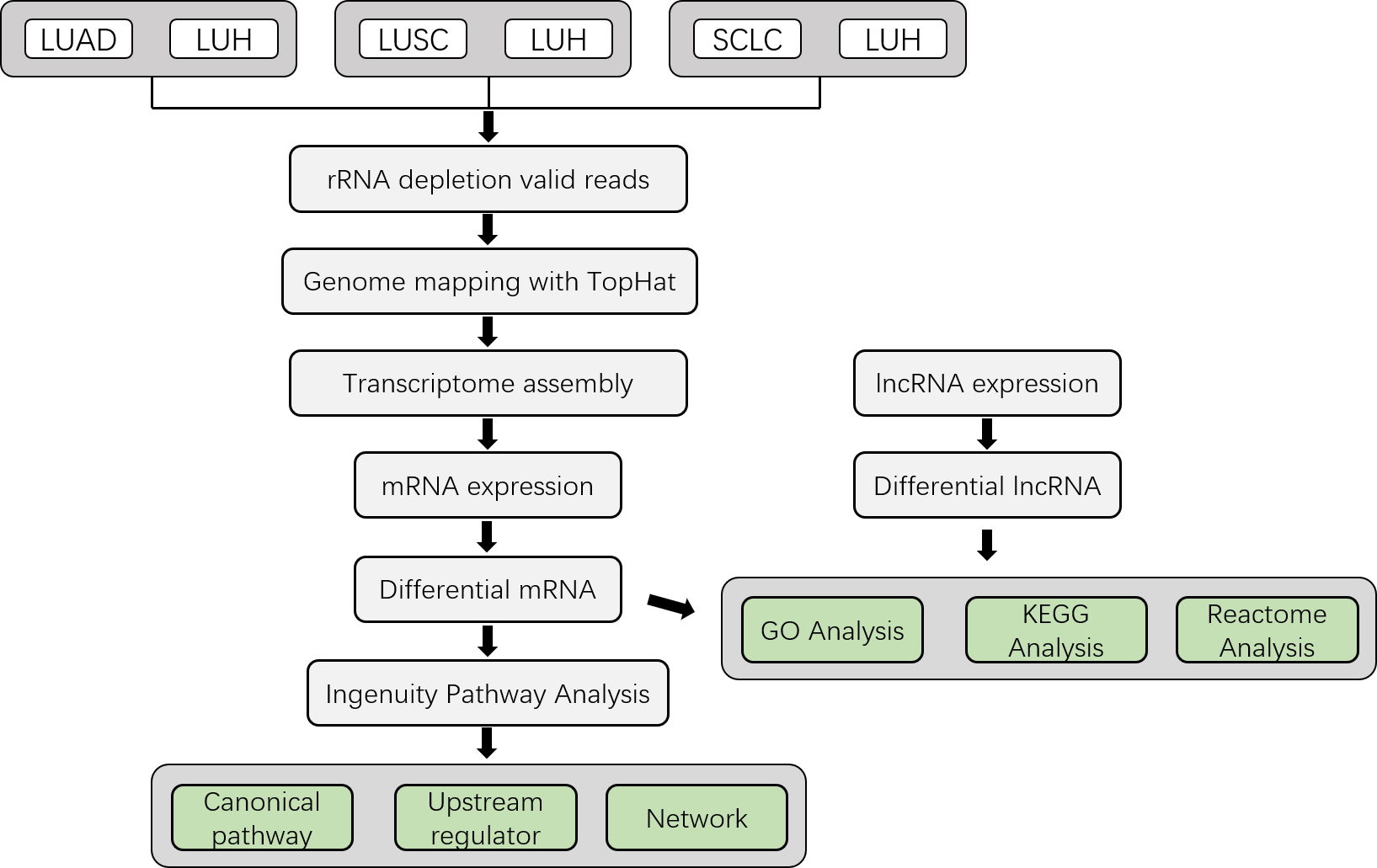

Supplement: Supplementary Figure 1 — The overall data analysis flow. LUAD, lung adenocarcinoma; LUSC, lung squamous cell carcinoma; SCLC, small cell lung cancer; LUH, lung hamartoma. [file Image_1.tif]

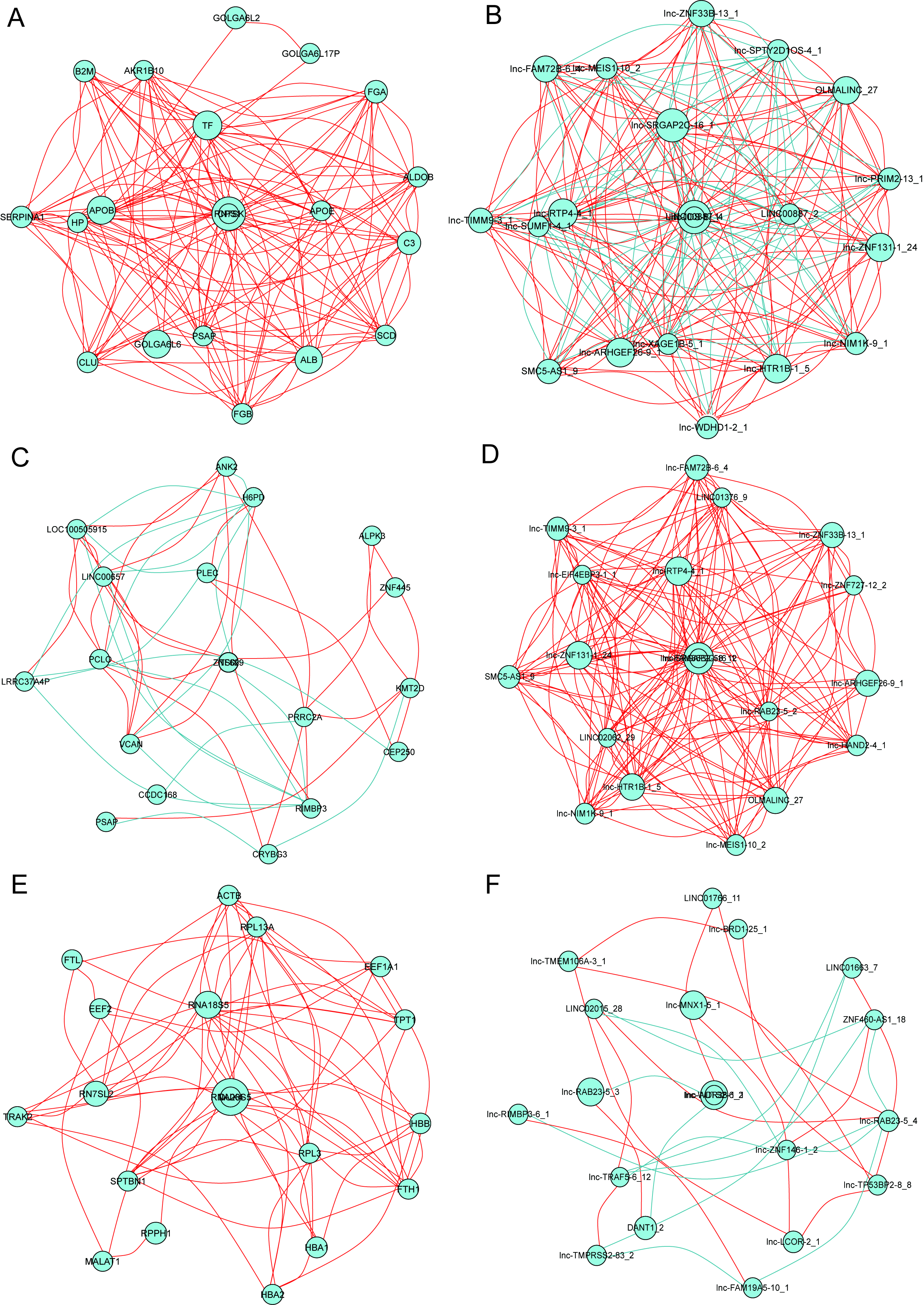

Supplement: Supplementary Figure 2 — The co-expression of differentially mRNAs and lncRNAs in the study. The co-expression of mRNAs (A) and lncRNAs (B) in LUAD and LUH. The co-expression of mRNAs (C) and lncRNAs (D) in LUSC and LUH; The co-expression of mRNAs (E) and lncRNAs (F) in SCLC and LUH. (LUAD, lung adenocarcinoma; LUSC, lung squamous cell carcinoma; SCLC, small cell lung cancer; LUH, lung hamartoma.) [file Image_2.tif]
