# Supplementary material for: Integrative Analyses of Circulating mRNA and lncRNA Expression Profile in Plasma of Lung Cancer Patients
Source: Front Oncol. 2022 Mar 31;12:843054. doi: 10.3389/fonc.2022.843054 (PMC9008738; doi:10.3389/fonc.2022.843054)
Supplement: Supplementary file 3 [file Table_1.docx]

Supplementary Table 1. The primers of qPCR in the study.

| Genes | Forward chain | Reverse chain |
| --- | --- | --- |
| ZNF891 | GCCTTCAACACGAGCTCTCA | AAGGGATGAGACCCCACTGA |
| ERCC4 | CAACGGCGGAGTTGTTTGAG | TCTCTGACTCGGGAAGGGTT |
| ZNF33A | AGCAACCTTGTCTCAGTGGG | TGGAAAGCTTTGGCTTGGGA |
| AFF2 | GTAGCAGTGGCAGCAACAAC | CATGATCTCGCAGAGGGGAC |
| lnc-FBXO33-2:3 | GGAAAGAGGTGCGACTGGAA | AGCATTGGGAGTCTTGTCGG |
| lnc-ALB-1:6 | GAGGACCTTTGTCCTGGTGG | GTGGAAAGCAGAGCACAAGC |
| lnc-DPH5-1:6 | CGCTTCTGCCCTTGCATTAC | AGTTCACATCTGCCGGGATG |
| LINC01376:1 | GGGGATCACAAACTCAGGGG | CTCCAAGTCAAGGGCTCCAG |
| EIF3I | AAGCATGTCCTCACTGGCTC | CCCAAAGTCAAAACCGCAGG |
| TRIM13 | GATCTGTGCTACTCGTGGGG | AAGGCATCCCTTTCCTGAGC |
| USP27X | TACATGCCCGCCATTTAGCA | TCATCACCTTTGCAGTGCCT |
| lnc-SLC9A3-6:1 | GCTGGTAAGAGCAGGTGGTT | GCTTTTTACATGGCAGCCCC |
| lnc-GPR27-5:1 | ACCTGAGGTTCAGGGACACT | ATTCCCTCCCATGTTCTGCC |
| lnc-PFKP-38:1 | CAAATGCCCTGTTTGCTGCT | GGGCCACAGAGAAGCAGATT |
| lnc-PGS1-1:12 | GAAAGGGAAGTGGGGCTACC | GGGTCTCCCCTCTGGAATCT |

(ZNF891, zinc finger protein 891; ERCC4, excision repair cross-complementation group 4; ZNF33A, zinc finger protein 33A; AFF2, AF4/FMR2 family, member 2; EIF3I, eukaryotic translation initiation factor 3, subunit I; TRIM13, tripartite motif containing 13, USP27X, ubiquitin specific peptidase 27, X-linked)
