# Supplementary material for: Integrative Analyses of Circulating mRNA and lncRNA Expression Profile in Plasma of Lung Cancer Patients
Source: Front Oncol. 2022 Mar 31;12:843054. doi: 10.3389/fonc.2022.843054 (PMC9008738; doi:10.3389/fonc.2022.843054)
Supplement: Supplementary file 4 [file Table_2.docx]

**Supplementary Table 2.** Clinical-pathological characteristics of the patients.

|  | LUAD | LUSC | SCLC | LUH |
| --- | --- | --- | --- | --- |
|  | n (%) | n (%) | n (%) | n (%) |
| Total, n | 11 | 3 | 2 | 4 |
| Age, mean (SD) | 67.3 (9.8) | 60.0 (4.6) | 54.5 (10.6) | 52.5 (6.7) |
| Sex (male) | 4 (36.4) | 3 (100) | 3 (100) | 0 (0) |
| TNM Stage |  |  |  |  |
| IA, IB | 3 (27.3) | 0 (0) | 1 (50) | NA |
| IIA, IIB | 5 (45.5) | 1 (33.3) | 1 (50) | NA |
| IIIA, IIIB, IIIC | 3 (27.3) | 2 (66.7) | 0 (0) | NA |
| IVA | 0 (0) | 0 (0) | 0 (0) | NA |
